# Directional and Strain-Specific Interaction Between *Lactobacillus plantarum* and *Staphylococcus aureus*

**DOI:** 10.3390/microorganisms12122432

**Published:** 2024-11-26

**Authors:** Sandeep Kondakala, Sunghyun Yoon, Soumana Daddy Gaoh, Ohgew Kweon, Seong-Jae Kim, Mark E. Hart

**Affiliations:** 1 Division of Microbiology, National Center for Toxicological Research, U.S. Food and Drug Administration, Jefferson, AR 72079, USA; sandeep.kondakala@fda.hhs.gov (S.K.); sunghyun.yoon@fda.hhs.gov (S.Y.); soumana.daddy-gaoh@fda.hhs.gov (S.D.G.); oh-gew.kweon@fda.hhs.gov (O.K.); seong-jae.kim@fda.hhs.gov (S.-J.K.); 2 Department of Microbiology and Immunology, University of Arkansas for Medical Sciences, Little Rock, AR 72205, USA

**Keywords:** antagonism, *Lactobacillus plantarum*, non-antibiotic biocontrol agent, proteomics, *Staphylococcus aureus* infection

## Abstract

The interaction between *Lactobacillus plantarum* and *Staphylococcus aureus* strains FRI-1169 and MN8, two original isolated strains from menstrual toxic shock syndrome (mTSS) cases, is a key focus for developing non-antibiotic strategies to control *S. aureus*-related infections. While the antagonistic effects of *Lactobacilli* species on *S. aureus* through mechanisms like organic acid and bacteriocin production are known, the molecular dynamics of these interactions remain underexplored. This study employs a proteomic approach to analyze the interactions between *L. plantarum* WCFS1 and *S. aureus* strains, FRI-1169 and MN8, during co-culture. We profiled differentially expressed proteins (DEPs) found in the spent media and cytosols of both bacteria, revealing distinct directional and strain-specific responses. The findings demonstrate that *L. plantarum* exerts a more pronounced effect on *S. aureus*, with more DEPs and upregulated proteins, while *S. aureus* showed fewer DEPs and more downregulated proteins. These strain-specific interactions highlight the complex metabolic and regulatory adjustments between these bacterial species. This research provides valuable insights into the molecular mechanisms of *Lactobacillus-S. aureus* antagonism and underscores the potential of proteomic analysis as a powerful tool for studying bacterial dynamics in co-culture systems.

## 1. Introduction

*Staphylococcus aureus* strains FRI-1169 and MN8 were originally isolated from menstrual toxic shock syndrome (mTSS) cases in the late 1970s [1,2,3,4]. These strains have become significant models for studying TSS due to their unique toxin production profiles [5]. While both strains produce toxic shock syndrome toxin-1 (TSST), *S. aureus* MN8, classified as a USA200 pulsed-field gel electrophoresis (PFGE) type, is known for being a low producer of α-toxin due to a nonsense mutation within the open reading frame of α-toxin, whereas *S. aureus* FRI-1169, though not part of the USA200 clonal type, is recognized for its high α-toxin production [5]. Despite the extensive knowledge of these strains, there remains a need for comprehensive genotypic and phenotypic evaluations, particularly concerning their roles in mTSS. The incidence of mTSS in the United States is rare, approximately 1 case/100,000 among menstrual age women (13–54 years of age) [6,7]. However, the fact that 5–10% of cases end in death warrants further efforts to find alternative approaches other than antibiotic therapy that can be developed for prevention and treatment before an event such as an epidemic with an antibiotic-resistant, TSST-1 producing strain of *S. aureus* occurs [8,9]. This becomes even more critical in light of the potential re-emergence of *S. aureus* mTSS clonal groups and the rise of antibiotic-resistant, TSST-1-producing strains of *S. aureus* [10,11,12].

The interaction between *Lactobacillus* species and *S. aureus* has attracted significant interest for its potential in non-antibiotic control of *S. aureus*-related infections, including toxic shock syndrome (TSS) and other serious conditions. *Lactobacillus* species, valued for their probiotic benefits, exhibit antagonistic effects on *S. aureus* through a variety of mechanisms, including the production of organic acids, bacteriocins, and hydrogen peroxide, as well as competition for nutrients [13,14]. Despite these findings, the precise molecular mechanisms underlying these interactions, especially at the proteomic level, are not yet fully understood. Uncovering these molecular dynamics is essential for the further development of *Lactobacillus* species as a biotherapeutic means for infection control.

Proteomic analysis has emerged as a crucial tool for elucidating bacterial interactions, providing detailed insights into cellular responses and metabolic adjustments. Previous research has often focused on isolated factors, such as the production of organic acids or bacteriocins, without exploring the broader, overall protein response as the result of interactions between *Lactobacillus* and *S. aureus*. Our group demonstrated that acid production by *Lactobacillus* species inhibits *S. aureus* MN8 but did not fully investigate the molecular mechanisms involved [15]. Additionally, while *L. plantarum* has well-established antagonistic properties, its specific effects on different *S. aureus* strains have not been extensively characterized. This study seeks to fill this gap by using proteomic data to explore interactions between *L. plantarum* and two *S. aureus* strains, FRI-1169 and MN8, with a particular focus on differentially expressed proteins (DEPs) and their functional roles in bacterial dynamics. This study utilizes a co-culture system that physically separates *S. aureus* from *L. plantarum* during growth and, thus, allows spent media produced by one species to interact with the other species without cell-to-cell contact. In addition, we were also able to isolate cells of each species and determine cytosolic differences as a result of this interaction.

In this study, we present the first comprehensive proteomic analysis of interactions between *L. plantarum* and *S. aureus*. By profiling DEPs in the spent media and cytosols of both *L. plantarum* and the two *S. aureus* strains during co-culture, this research sheds light on the metabolic and regulatory pathway changes in response to each other. The findings presented enhance our understanding of *Lactobacillus*-*S. aureus* antagonism and demonstrate the utility of proteomic analysis as a powerful tool for studying complex bacterial dynamics in co-culture systems.

## 2. Materials and Methods

### 2.1. Bacterial Strains, Strain Maintenance, and Growth Conditions

*Staphylococcus aureus* clinical strains FRI-1169 (1980 WI; NCH 240) and MN8 (1980 MN; NCH 245) were isolated from cases of mTSS and were provided by P.D. Fey at the University of Nebraska Medical Center and were confirmed to produce toxic shock syndrome toxin-1 (TSST-1) by western analysis. Strains were maintained as frozen (−80 °C) stocks in brain heart infusion (BHI; Difco Laboratories, Detroit, MI, USA) broth containing 25% (*w*/*v*) glycerol and streaked for isolation on either tryptic soy broth (TSB; Difco Laboratories) or TSB containing 1.5% agar. *Lactobacillus plantarum* WCFS1 (HLH 10) was obtained from Wageningen Center for Food Sciences, Wageningen, Netherlands, and maintained as a frozen stock in Lactobacilli deMan, Rogosa and Sharpe (MRS) broth (Difco Laboratories) containing 10–15% (*w*/*v*) glycerol and streaked for isolation on MRS containing 1.5% agar. While *Lactobacillus* species, namely *L. crispatus*, *L. gasseri*, *L. iners*, and *L. jensenii* are the predominant members of this genus found in most vaginal tracts of healthy women [16,17,18], *L. plantarum* can be found in the urogenital tract of women but its numbers appear to be low [16,17,19], thus leaving questions as to its role as a member of the vaginal microbiota. Nevertheless, our decision to utilize *L. plantarum* WCFS1 [20] for most of our studies thus far, was made primarily due to convenience. *Lactobacillus plantarum* WCFS1 was the first of the genus *Lactobacillus* to have its genome completely sequenced and annotated [21,22], thus facilitating the molecular studies to isolate and clone important *L. plantarum* genetic determinants for the purpose of cloning the lysostaphin gene (*end*) into *L. plantarum* WCFS1 [20].

### 2.2. Co-Culture and Sample Preparation

Cells of *L. plantarum* WCFS1 and *S. aureus* strains FRI-1169 and MN8 were cultured overnight (15–18 h) in MRS and TSB media, respectively, using a flask to a medium volume of 2.5. A 1 mL aliquot of each overnight culture was transferred to a sterile microcentrifuge tube, washed twice with 1 mL of sterile, deionized water, and once with 1 mL of buffered, Hart modified genital tract secretion (HmGTS) medium [23]. The medium was made without the inclusion of bovine serum albumin to facilitate bacterial protein identification by mass spectrophotometry. The cells were then suspended in 1 mL of HmGTS media, and optical density (OD) readings were determined for each culture spectrophotometrically at 550 nm. Optical density values were used to dilute cultures to approximately 1 × 10^7^ colony-forming units (cfu)/mL using a previously determined standard curve of cfu as a function of OD. Two ml of *S. aureus* cell suspensions were then aseptically pipetted into each well of a six-well BD Falcon cell culture plate (Becton Dickinson, Franklin Lakes, NJ, USA). Cup-shaped inserts with 0.4 µm filters were aseptically placed into each well, and 2 mL of *L. plantarum* cell suspensions were aseptically pipetted into each insert. Control samples consisted of 2 mL cell suspensions of either *S. aureus* or *L. plantarum* aseptically pipetted into both the well and insert. Plates were incubated under stationary, aerobic conditions at 37 °C prior to harvesting both well and insert contents at 8 and 24 h of growth. Viable cell counts were determined using a portion of each of the cell suspensions at 0, 8, and 24 h using 10-fold serial dilutions and plating on mannitol salt agar (MSA), which is selective for *S. aureus,* and Rogosa agar (RA), which is selective for *L. plantarum*. The remaining volume of each cell suspension was centrifuged (12,000× *g*, for 10 min at 4 °C) to separate spent media from cells. Spent media were filter-sterilized into 15 mL polypropylene, screw-capped, conical centrifuge tubes using 0.2 μm syringe filters. Portions of each filter-sterilized spent medium were pipetted into 4 mL capacity, Centricon tubes (Centricon Ultracel YM-3 filters with 3000 MWCO, Millipore Corporation, Bedford, ME, USA) and centrifuged (7500× *g*) at 4 °C for 1 h. The retentates were recovered and stored at –80 °C until needed. Cell pellets from the first centrifugation were washed one time in one ml of TE (10 mM and 1 mM EDTA) buffer (pH 8.0), suspended in 0.4 mL of TE, and transferred to FastPrep blue tubes (Qbiogene, Irvine, CA, USA) containing acid-washed and RNase free, 0.1 mm silica beads and lysed using the FP120 reciprocator (Qbiogene) with a setting of 6 m/second for 40 s. Cell lysates were immediately cooled on ice for 15 min and then subjected to centrifugation (10,000× *g* for 1 min at 4 °C). Supernatants were recovered and stored at –80 °C.

### 2.3. Protein Quantification and Sample Quality Assessment

The protein concentration for each sample was determined using the bicinchoninic acid (BCA) assay (Pierce Biotechnology, Waltham, MA, USA) following the manufacturer’s instructions. The quality of the processed samples was assessed using NuPAGE Tris-Bis mini gels (4–12% gradient) according to the manufacturer’s instructions (Invitrogen Life Technologies, Carlsbad CA, USA).

### 2.4. Protein Digestion and Peptide Preparation

An aliquot of the protein extract (50 µg) was reduced with 12 mM dithiothreitol (DTT) at room temperature for 1 h. This was followed by alkylation with 15 mM iodoacetamide (IAA) at room temperature for 1 h. Trypsin (sequencing grade, Promega, Madison, WI, USA) was added at a 1:20, enzyme to substrate dilution, and the digestion was carried out overnight at 37 °C. The digested peptides were acidified with 1% formic acid and desalted using C18 solid-phase extraction columns (Waters, Milford, MA, USA). Peptides were then dried in a vacuum concentrator and stored at –80 °C until mass spectrometry analysis.

### 2.5. Mass Spectrometry Analysis

For all samples, 1 µg was analyzed by nano LC/MS with a Waters M-class HPLC system interfaced with an Orbitrap Exploris 480 Mass Spectrometer (ThermoFisher, Walthma, MA, USA). For generation of the DIA Chromatogram Library, six gas-phase fractions (GPF) injections were acquired for six ranges: 396 to 502, 496 to 602, 596 to 702, 696 to 802, 796 to 902, and 896 to 1002. Sequentially, full scan MS data (60,000 FWHM resolution) was followed by 26 × 4 *m*/*z* precursor isolation windows, another full scan and 26 × 4 *m*/*z* windows staggered by 2 *m*/*z*. The products were acquired at 30,000 FWHM resolution. The automatic gain control (AGC) target was set to 1 × 10^6^ for both full MS and product ion data. The maximum ion inject time (IIT) was set to 50 ms for full MS and “dynamic” mode for products with nine data points required across the peak and the NCE was set to 30.

For sample analysis, full scan MS data (60,000 FWHM resolution) from *m*/*z* 385 to 1015 was followed by 61 × 10 *m*/*z* precursor isolation windows, another full scan from *m*/*z* 385 to 1015 was followed by 61 × 10 *m*/*z* windows staggered by 5 *m*/*z*. The products were acquired at 15,000 FWHM resolution. The maximum ion inject time (IIT) was set to 50 ms for full MS and “dynamic” mode for products with nine data points required across the peak and the NCE was set to 30. An injection of the pool was included at the start and end of the batch.

Data were processed using Scaffold for data-independent acquisition (Scaffold DIA 3.2.1; Proteome Software) which incorporated the following steps: raw MS files were converted to mzML (ProteoWizard 3.0.19254), including deconvolution of staggered windows and final conversion to DIA format. The alignment was based on retention times and used to create the custom DIA-based chromatogram libraries (ELIB); we searched data using the Prosit library for data-dependent acquisition (DLIB) and the chromatogram/reference library. Filtering of the database was performed using Percolator at 1% peptide false discovery rate (FDR). Peak areas for detected peptides were calculated using Encyclopedia (0.9.6). For each peptide, the five highest-quality fragment ions were selected for quantification. The data was searched with the following parameters: Enzyme: Trypsin, Databases: Uniprot *L. plantarum* and *S. aureus*, Fixed Modification: Carbamidomethyl (C); Precursor Mass Tolerance, Fragment Mass Tolerance and Library Fragment Tolerance were set at 10 ppm, Peptide and Protein FDR set at 0.01, Peptide Length: 6–30 AA, Maximum Missed Cleavages and Minimum Peptides set to 1 and Peptide charge set at 2–3.

### 2.6. Bioinformatic Analysis

Differentially expressed proteins (DEPs) were identified by filtering the proteomic data with a fold ratio of ≥ 2.0 based on normalized protein intensity values. The identified differentially expressed proteins (DEPs) were analyzed for Cluster of Orthologous Groups (COG) for functional categories, and the Kyoto Encyclopedia of Genes and Genomes (KEGG) database (http://www.genome.jp/kegg/ accessed on 7 August 2024) was used for pathway mapping [24]. In this study, the hypergeometric test was used to estimate the probability of finding a specific number of proteins associated with a particular function or pathway within the sample, taking into account the total number of proteins with that function in the entire population and the size of the sample. Figures and statistical analyses were generated using an in-house Python (3.10.0) script.

## 3. Results and Discussion

By enhancing our understanding of the mechanisms underlying *Lactobacillus*–*S. aureus* interactions, new avenues for the biocontrol of *S. aureus* using non-antibiotic methods that do not harm bacterial ecosystems are being explored [25,26,27]. The interaction between *L. plantarum* and *S. aureus* has been extensively studied to understand their dynamics [15,20,28]. Functional genomic approaches, such as transcriptomic and proteomic analyses, are considered powerful tools for investigating *Lactobacillus*–*S*. *aureus* interactions in terms of antagonistic mechanisms of *Lactobacillus* and cellular responses of *S. aureus*. To our knowledge, this study is the first to demonstrate that these bacterial interactions can be effectively analyzed through proteome analysis. We profiled the proteins in the spent media and cytosols of *S. aureus* strains FRI-1169 and MN8, as well as *L. plantarum* WCFS1, during co-culture, focusing on the proteins that were differentially expressed. Identifying these DEPs not only deepens our understanding of the specific interactions between these bacterial species but also underscores the potential of proteome analysis as a method for studying bacterial dynamics.

### 3.1. Interaction between L. plantarum WCFS1 and S. aureus FRI-1169

#### 3.1.1. Comparative Analysis of Proteins from *L. plantarum* WCFS1 During Co-Culture with *S. aureus* FRI-1169

**Figure 1:**
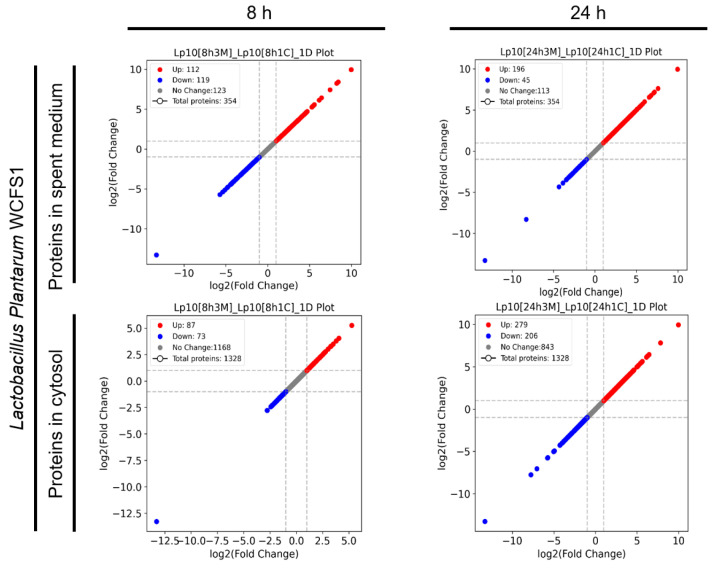
A comparative analysis of protein expression profiles in the spent medium and cytosol of *L. plantarum* WCFS1 during co-culture with *S. aureus* FRI-1169 at 8 h and 24 h.

To assess the response of *L. plantarum* WCFS1 during co-culture with *S*. *aureus* FRI-1169, we examined the protein expression profiles in both the spent medium and cellular cytosol of *L. plantarum* WCFS1 (Figure 1). Compared to control cultures, a total of 354 proteins were identified in the spent medium at 8 and 24 h, with 231 and 241 proteins differentially expressed at these time points, respectively. In the cytosol, 1328 proteins were identified, of which 160 and 485 proteins were differentially expressed at 8 and 24 h. Notably, many of the DEPs were upregulated, suggesting an active metabolic and cellular response by *L*. *plantarum* to *S*. *aureus*. This upregulation may reflect mechanisms that allow *L*. *plantarum* to enhance its antagonistic activity or adapt to environmental changes in the co-culture, highlighting the dynamic nature of bacterial interactions and the potential of *L*. *plantarum* to reinforce metabolic pathways to either sustain its own growth or inhibit *S*. *aureus*.

**Figure 2:**
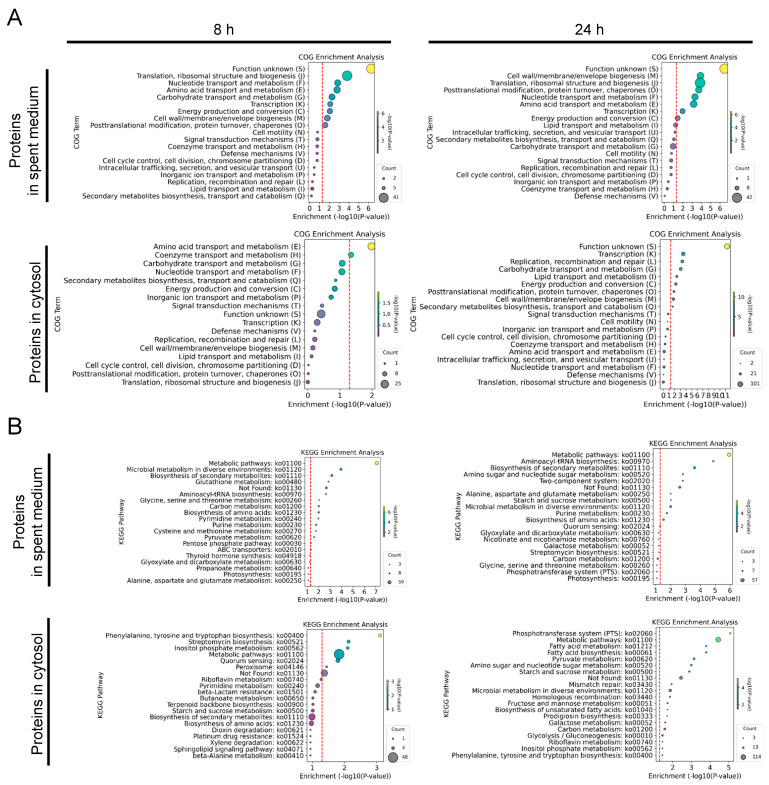
Functional and pathway analysis of proteins of *L. plantarum* WCFS1 during co-culture with *S. aureus* FRI-1169. COG enrichment analysis of DEPs in the spent medium and cytosol of *L. plantarum* WCFS1 at 8 and 24 h (**A**). KEGG enrichment analysis of proteins of *L. plantarum* WCFS1 at 8 and 24 h (**B**). The size of each circle represents the number of proteins. The hue of the circles symbolizes −log10(*p*-value) values. The red dashed lines represent the value of −log10(*p*-value = 0.05).

To further elucidate the biological processes and pathways involved in the interaction between *L. plantarum* WCFS1 and *S. aureus* FRI-1169, COG, and KEGG enrichment analyses were performed on the identified proteins from *L. plantarum* WCFS1. The COG enrichment analysis of DEPs from both the spent medium and cytosol at 8 and 24 h highlighted several key functional categories (Figure 2A). In the COG enrichment analysis of the DEPs in the spent medium, prominent among these were “translation, ribosomal structure, and biogenesis (J)”, “nucleotide transport and metabolism (F)”, “amino acid transport and metabolism (E)”, and “cell wall/membrane/envelope biogenesis (M)”, indicating active engagement by *L. plantarum* in essential metabolic and structural processes throughout the co-culture period. On the other hand, in the COG enrichment analysis of the DEPs in the cytosol, at 8 h, the prominent pathways of *L. plantarum* primarily were nutrient acquisition and metabolic activity, with significant enrichment in “amino acid transport and metabolism (E)” and “coenzyme transport and metabolism (H)”. By 24 h, the metabolism shifted toward processes related to gene regulation and genomic stability, with enrichment observed in “transcription (K)” and “replication, recombination, and repair (L)”. Additionally, consistent enrichment in “carbohydrate transport and metabolism (G)” throughout the experiment, along with later shifts toward “lipid transport and metabolism (I)” and “energy production and conversion (C)”, reflect the ongoing metabolic adjustments made by *L. plantarum* WCFS1 in response to co-culture with *S. aureus* FRI-1169. These observations suggest that *L. plantarum* dynamically regulates its metabolic and structural processes, initially prioritizing nutrient acquisition and later focusing on cellular maintenance and genomic integrity as the co-culture progresses. This indicates a well-coordinated response that could allow *L. plantarum* to adapt to the environmental conditions presented by the presence of *S. aureus*.

The KEGG enrichment analysis of proteins from the spent medium of *Lactobacillus plantarum* WCFS1 during co-culture with *Staphylococcus aureus* FRI-1169 revealed key metabolic and biosynthetic activities at both 8 and 24 h (Figure 2B). In the spent medium, at 8 h, the prominently enriched pathways included “metabolic pathways”, “aminoacyl-tRNA biosynthesis”, and “biosynthesis of secondary metabolites”, suggesting that *L. plantarum* was actively engaged in core metabolic and biosynthetic processes early in the co-culture. By 24 h, these pathways remained highly enriched, with additional enrichment observed in pathways such as “amino sugar and nucleotide sugar metabolism” and “two-component system”, reflecting ongoing metabolic activities and the emergence of regulatory responses as the co-culture progressed.

In the cytosol, *L. plantarum* WCFS1 exhibited significant enrichment at 8 h in pathways related to “phenylalanine, tyrosine, and tryptophan biosynthesis”, “streptomycin biosynthesis”, and “inositol phosphate metabolism”, indicating robust early biosynthetic activity. Furthermore, enrichment in pathways like “quorum sensing” and “peroxisome” pointed to broader regulatory mechanisms being activated in response to *S. aureus*. By 24 h, the enrichment of the top 20 pathways increased significantly, suggesting a more dynamic and adaptive metabolic response in the later stages of co-culture. These results highlight how *L. plantarum* adjusts its metabolic and regulatory pathways over time, likely in response to interactions with *S. aureus*, underscoring the complexity of the bacterial interactions.

To analyze the cellular response between *L. plantarum* WCFS1 and *S. aureus* FRI-1169 and between *L. plantarum* WCFS1 and *S. aureus* MN8, we examined the expression of proteins related to antibiotic resistance, heavy metal resistance, and virulence factors (Supplementary Tables S1 and S2). These functions are often used to assess bacterial status in unfavorable environments, indicating how bacteria adapt, survive, and potentially thrive despite stressors. Additionally, other proteins with notable upregulation were analyzed for their roles during co-culture. Understanding these protein expressions would help elucidate the mechanisms employed by each bacterium in response to the challenging environment.

In the genome of *L. plantarum* WCFS1, 29 heavy metal resistance genes were identified, with 20 proteins detected, 10 of which were upregulated. Proteins like CopY/TcrY and CopB may regulate internal conditions [28], while YieF and ChrR are associated with oxidative stress management [29], although these responses likely reflect regular metabolic activities rather than stress responses. Among the 37 identified virulence factors, 24 proteins were detected, with 8 showing upregulation. The Clp proteins are involved in maintaining protein quality [30,31,32].

#### 3.1.2. Comparative Analysis of Proteins from *S. aureus* FRI-1169 During Co-culture with *L. plantarum* WCFS1

**Figure 3:**
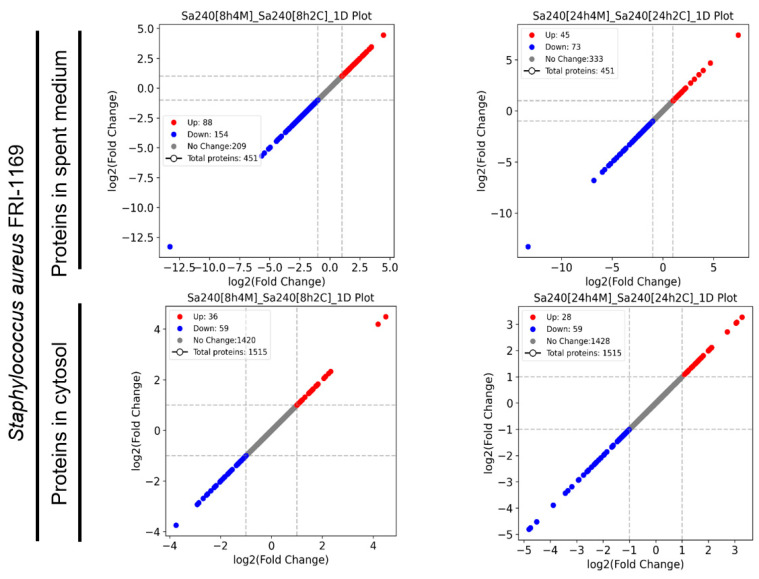
A comparative analysis of protein expression profiles in the spent medium and cytosol of *S. aureus* FRI-1169 during co-culture *L. plantarum* WCFS1 at 8 h and 24 h.

The growth inhibition of *S. aureus* FRI-1169 by *L. plantarum* WCFS1 was supported by proteomic evidence. To investigate the response of *S. aureus* FRI-1169 during co-culture with *L. plantarum* WCFS1, the protein expression profiles from both the spent medium and cellular cytosol were analyzed (Figure 3). At 8 and 24 h, 451 proteins were identified in the spent medium, with 242 and 118 proteins differentially expressed at these respective time points. Similarly, 1515 cytosolic proteins were identified, with differential regulation observed in 95 proteins at 8 h and 87 at 24 h.

**Figure 4:**
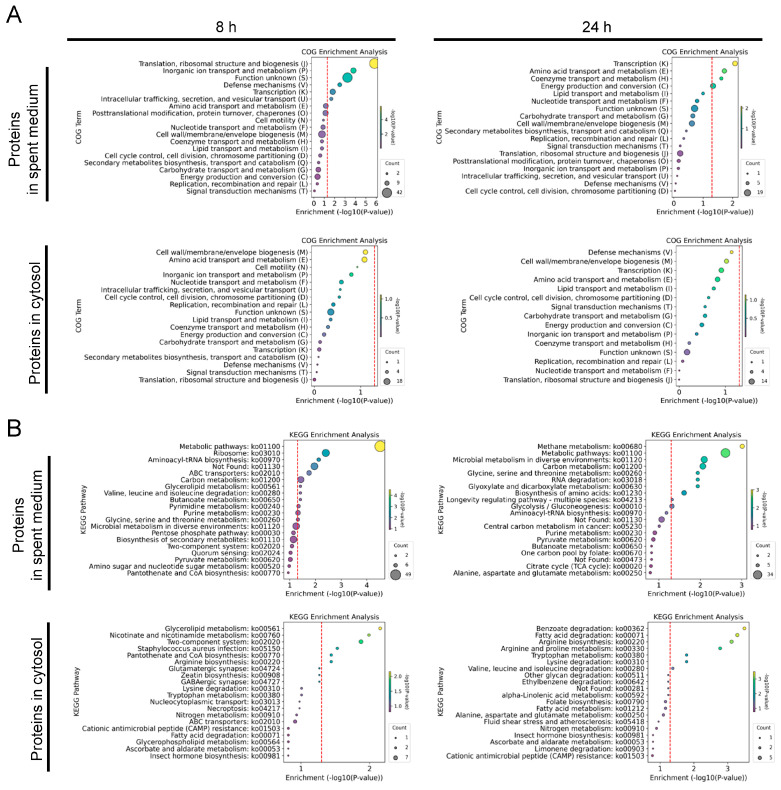
Functional and pathway analysis of proteins of *S. aureus* FRI-1169 during co-culture with *L. plantarum* WCFS1. COG enrichment analysis of DEPs in the spent medium and the cytosol of *S. aureus* FRI-1169 at 8 and 24 h (**A**). KEGG enrichment analysis of proteins of *S. aureus* FRI-1169 at 8 and 24 h (**B**). The size of each circle represents the number of proteins. The hue of the circles symbolizes −log10(*p*-value) values. The red dashed lines represent the value of −log10(*p*-value = 0.05).

COG enrichment analysis of DEPs from the spent medium revealed dynamic changes over time (Figure 4A). At 8 h, there was significant enrichment in “translation, ribosomal structure and biogenesis (J)”, “inorganic ion transport and metabolism (P)”, and “defense mechanisms (V)”, suggesting that *S. aureus* was mounting early responses focused on protein synthesis, ion regulation, and managing stress, likely in reaction to the acidic environment created by *L. plantarum*. By 24 h, the emphasis shifted towards categories like “transcription (K)”, “amino acid transport and metabolism (E)”, and “coenzyme transport and metabolism (H)”, indicating an adaptive response involving gene regulation and nutrient metabolism as *S. aureus* adjusted to the co-culture. In the cytosol, no significant enrichment was detected.

KEGG enrichment analysis of DEPs in the spent medium at 8 h showed significant enrichment in pathways related to protein synthesis and general metabolism, including “metabolic pathways”, “ribosome”, and “aminoacyl-tRNA biosynthesis” (Figure 4B). Other pathways, such as “ABC transporters” and “glycerolipid metabolism”, were enriched, indicating adaptations in nutrient uptake and lipid processing. By 24 h, continued enrichment in pathways such as “methane metabolism” and “RNA degradation” highlighted sustained metabolic activity (Figure 4B). In the cytosol, at 8 h, enrichment in pathways like “glycerolipid metabolism” and “two-component system” pointed to early metabolic adaptations. Pathways like “pantothenate and CoA biosynthesis” and “arginine biosynthesis” suggest ongoing nutrient metabolism and homeostasis, particularly in response to the acidic conditions imposed by *L. plantarum*. By 24 h, amino acid, and lipid metabolism remained central, with significant enrichment in “fatty acid degradation” and “arginine biosynthesis”, reflecting further adaptation and stress resistance mechanisms.

Notably, *S. aureus* FRI-1169 exhibited upregulation of proteins related to antibiotic resistance and stress response. Among the 23 antibiotic resistance genes identified, 12 corresponding proteins were detected during co-culture, including three upregulated proteins: MgrA, RpoB2, and VanR. MgrA, a global transcriptional regulator, modulates resistance mechanisms and is in involved with virulence gene regulation [33,34,35]. RpoB2, a subunit of RNA polymerase, likely plays a role in regulating gene expression under these stress conditions [36], while VanR, a regulator of vancomycin resistance, may help maintain cell wall integrity [37]. Additionally, out of the 123 identified virulence factors, 76 corresponding proteins were detected, with 16 upregulated, including ClpB, ClpC, GroL, and others involved in stress responses and protein quality control [38,39]. These proteins likely help *S. aureus* manage the harsh conditions of the co-culture, ensuring its survival despite the antagonistic effects of *L. plantarum*.

### 3.2. Interaction Between L. plantarum WCFS1 and S. aureus MN8

#### 3.2.1. Comparative Analysis of Proteins from *L. plantarum* WCFS1 During Co-Culture with *S. aureus* MN8

**Figure 5:**
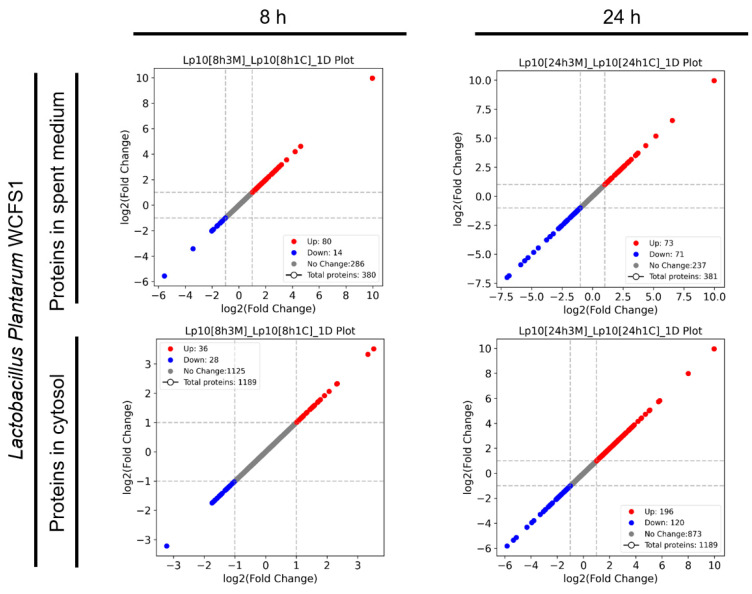
A comparative analysis of protein expression profiles in the spent medium and cytosol of *L. plantarum* WCFS1 during co-culture with *S. aureus* MN8 at 8 h and 24 h.

To evaluate the response of *L. plantarum* WCFS1 when co-cultured with *S. aureus* MN8, we analyzed the protein expression profiles from both the spent medium and cytosol (Figure 5). At 8 and 24 h, 380 and 381 proteins were identified in the spent medium, respectively, with 94 and 144 proteins showing differential expression at these time points. In the cytosol, 1189 proteins were detected, with 64 proteins differentially expressed at 8 h and 316 at 24 h. This differential regulation reflects an adaptive response by *L. plantarum* in co-culture with *S. aureus* MN8, including shifts in metabolic processes and cellular activities over time.

**Figure 6:**
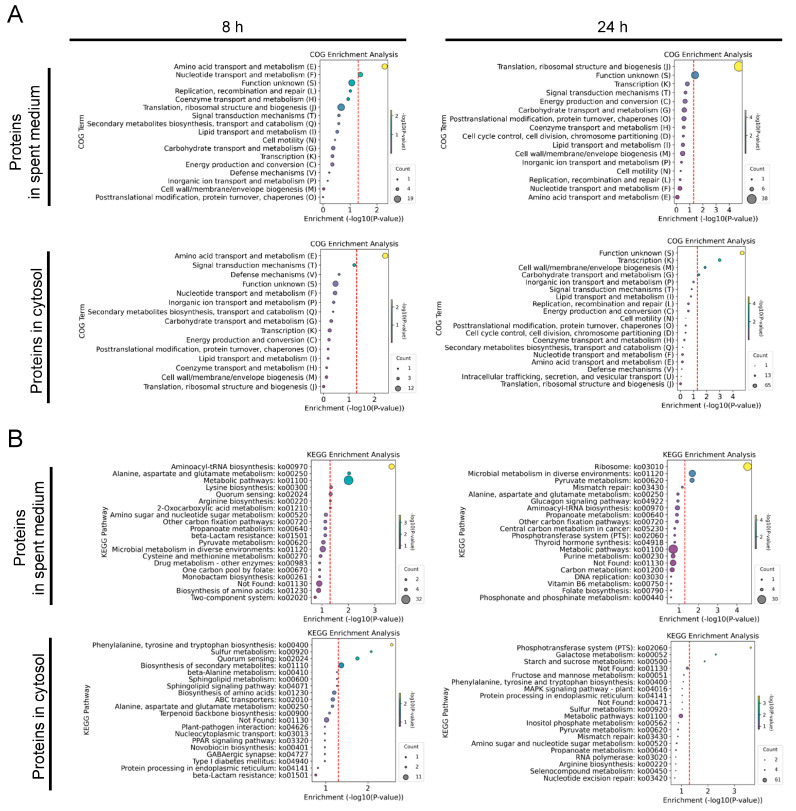
Functional and pathway analysis of proteins from *L. plantarum* WCFS1 during co-culture with *S. aureus* MN8. COG enrichment analysis of DEPs in the spent medium and cytosol of *L. plantarum* WCFS1 at 8 and 24 h (**A**). KEGG enrichment analysis of proteins from *L. plantarum* WCFS1 at 8 and 24 h (**B**). The size of each circle represents the number of proteins. The hue of the circles symbolizes −log10(*p*-value) values. The red dashed lines represent the value of −log10(*p*-value = 0.05).

COG enrichment analysis of the DEPs revealed dynamic functional responses in *L. plantarum* during co-culture (Figure 6A). At 8 h, key categories such as “amino acid transport and metabolism (E)” and “nucleotide transport and metabolism (F)” were enriched, indicating early metabolic activity. By 24 h, there was a notable shift toward “translation, ribosomal structure, and biogenesis (J)”, suggesting an increased focus on protein synthesis as the co-culture progressed. In the cytosol, early enrichment at 8 h in categories such as “amino acid transport and metabolism (E)” and “signal transduction mechanisms (T)” indicated active nutrient acquisition and cellular signaling. By 24 h, the focus shifted to “transcription (K)” and “cell wall/membrane/envelope biogenesis (M)”, reflecting gene regulation and structural functions in response to the ongoing interaction.

KEGG enrichment analysis of the DEPs further highlighted the metabolic adjustments made by *L. plantarum* (Figure 6B). At 8 h, pathways like “aminoacyl-tRNA biosynthesis”, “alanine, aspartate, and glutamate metabolism”, and “lysine biosynthesis” were enriched, pointing to active protein synthesis and amino acid metabolism. By 24 h, pathways involved in “ribosome” and “pyruvate metabolism” were prominent, reflecting continued protein synthesis and energy production. In the cytosol, early enrichment in “phenylalanine, tyrosine, and tryptophan biosynthesis” and “sulfur metabolism” suggested normal metabolic activity, while by 24 h, enrichment in the “phosphotransferase system (PTS)” and “starch and sucrose metabolism” indicated ongoing carbohydrate metabolism.

Out of the 29 heavy metal resistance genes of *L. plantarum* WCFS1, 18 corresponding proteins were detected during co-culture with *S. aureus* MN8, with 5 upregulated, including GalE, RecG, and Rrp2. Although no heavy metal stress was present, the upregulation suggests cellular processes related to environmental adaptation, with GalE maintaining cellular functions [39] and RecG and Rrp2 supporting cellular maintenance [40,41]. Among 37 virulence factors identified, 24 corresponding proteins were detected, with 8, such as ClpE, ClpB, Tuf, GroL, PpiB, RfbB, TagH, and KatA, upregulated [42].

#### 3.2.2. Comparative Analysis of Proteins from *S. aureus* MN8 During Co-Culture with *L. plantarum* WCFS1

**Figure 7:**
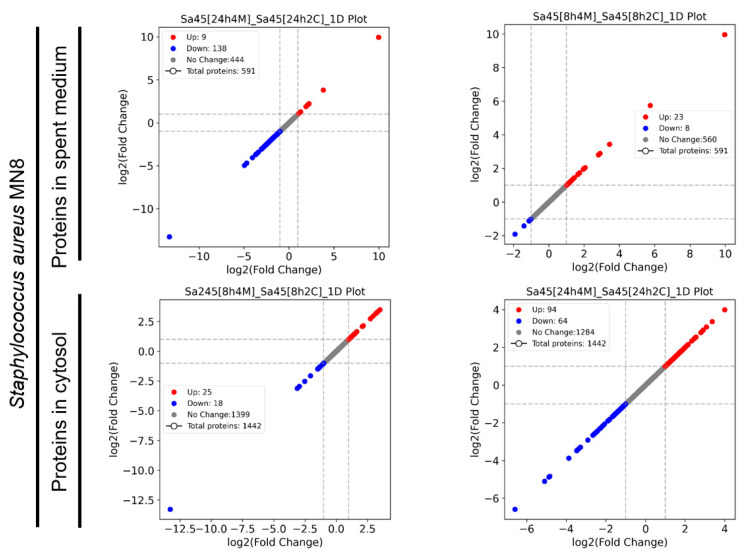
A comparative analysis of protein expression profiles in the spent medium and cytosol of *S. aureus* MN8 during co-culture with *L. plantarum* WCFS1at 8 h and 24 h.

To evaluate how *S. aureus* MN8 responds to co-culture with *L. plantarum* WCFS1, the protein expression profiles in both the spent medium and cellular cytosol were analyzed. As shown in Figure 7, protein expression in the spent medium of *S. aureus* MN8 at 8 and 24 h was compared to that in the control culture. A total of 591 proteins were identified at both time points, with 147 proteins differentially expressed at 8 h and 31 proteins at 24 h. In the cytosol, 1442 proteins were identified, with differential regulation observed in 43 proteins at 8 h and 158 proteins at 24 h.

**Figure 8:**
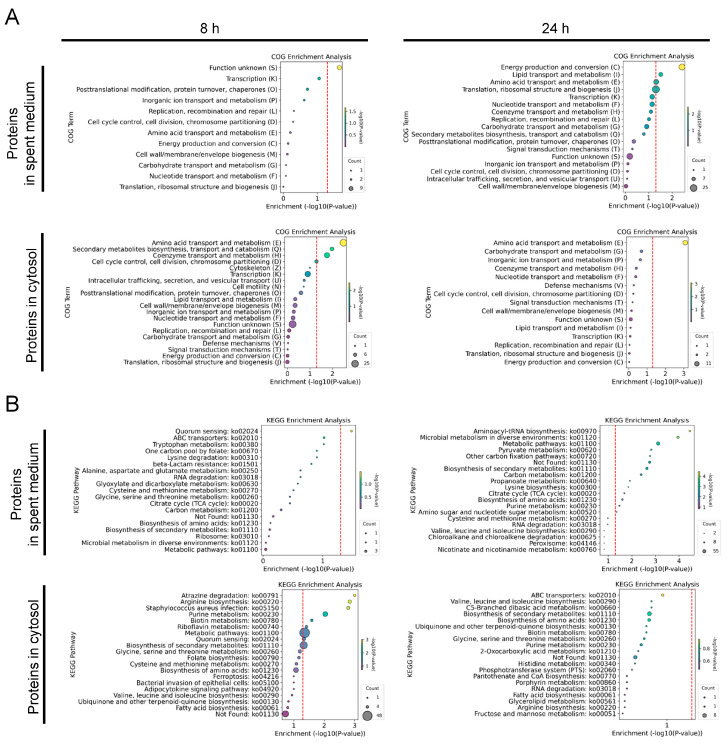
Functional and pathway analysis of proteins of *S. aureus* MN8 during co-culture with *L. plantarum* WCFS1. COG enrichment analysis of DEPs in the spent medium and the cytosol of *S. aureus* MN8 at 8 and 24 h (**A**). KEGG enrichment analysis of proteins of *S. aureus* MN8 at 8 and 24 h (**B**). The size of each circle represents the number of proteins. The hue of the circles symbolizes −log10(*p*-value) values. The red dashed lines represent the value of −log10(*p*-value = 0.05).

COG enrichment analysis (Figure 8A) revealed that at 24 h, DEPs in the spent medium of *S. aureus* MN8 showed enrichment in categories such as “energy production and conversion”, “lipid transport and metabolism (I)”, and “amino acid transport and metabolism”. These enrichments indicate active metabolic processes essential for cellular adaptation to environmental changes, potentially including adjustments to pH fluctuations caused by *L. plantarum*. In the cytosolic proteins, *S. aureus* MN8 exhibited early metabolic activity at 8 h, with enrichment in “amino acid transport and metabolism”, “secondary metabolites biosynthesis, transport, and catabolism (Q)”, and “coenzyme transport and metabolism (H)”. By 24 h, the focus remained on “amino acid transport and metabolism”, suggesting continued engagement in essential metabolic processes.

KEGG enrichment analysis of the DEPs (Figure 8B) highlighted key pathways in the spent medium at 8 h, including significant enrichment in “quorum sensing”, indicating that bacterial communication was actively occurring early in the co-culture. By 24 h, pathways such as “aminoacyl-tRNA biosynthesis”, “microbial metabolism in diverse environments”, and “metabolic pathways” were enriched, reflecting continued protein synthesis, metabolic activity, and microbial interactions. Further enrichment in “pyruvate metabolism”, “biosynthesis of secondary metabolites”, and “citrate cycle (TCA cycle)” suggested ongoing energy production and carbon metabolism as *S. aureus* MN8 adapted to the co-culture environment. In the cytosol, enrichment at 8 h in pathways like “atrazine degradation”, “arginine biosynthesis”, and “*Staphylococcus* aureus infection” pointed to roles in nitrogen processing, amino acid biosynthesis, and virulence mechanisms. Enrichment in “purine metabolism”, “riboflavin metabolism”, and “quorum sensing” at 8 h indicated nucleotide metabolism and bacterial communication during the early stages of interaction.

In the genome of *S. aureus* MN8, 23 antibiotic resistance genes, 36 heavy metal resistance genes, and 116 virulence factor genes were identified, with several corresponding proteins detected during co-culture with *L. plantarum* WCFS1. Among these, the virulence factors SdrF, UreC, UreG, and FabZ were upregulated. UreC and UreG, components of the urease system, likely played a role in maintaining pH by converting urea into ammonia, helping *S. aureus* neutralize the acidic environment induced by *L. plantarum* [43].

### 3.3. Differential Expression of Proteins from L. plantarum WCFS1 During Co-Culture

#### 3.3.1. Protein Expression Profiles of *L. plantarum* WCFS1 During Co-Culture

**Figure 9:**
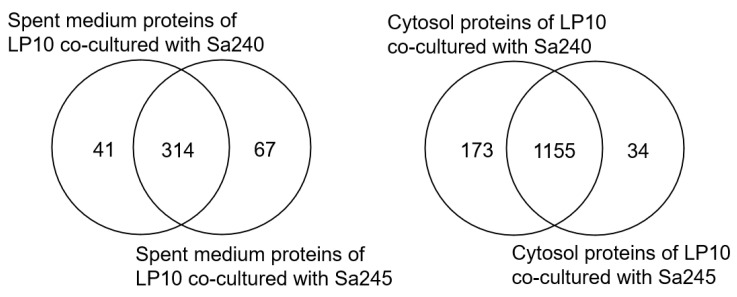
Venn diagram analysis of DEPs in the spent medium (**left**) and the cytosol (**right**) of *L. plantarum* WCFS1 during co-culture with *S. aureus* FRI-1169 and *S. aureus* MN8.

We assessed whether the protein expression profiles of *L. plantarum* WCFS1 are consistent when co-cultured with two different strains of *Staphylococcus aureus*—FRI-1169 and MN8 (Supplementary Table S3). The Venn diagram (Figure 9) illustrates the comparison of differentially expressed proteins between the two co-cultures. In the spent medium, 41 and 67 proteins (25.7%) were differentially expressed in the co-cultures with *S. aureus* FRI-1169 and MN8, respectively, while 314 proteins were expressed in common. In the cell lysate, 173 and 34 cytosolic proteins (15.1%) were differentially expressed, with 1155 proteins shared between the two conditions. These results demonstrate that the protein expression profiles of *L. plantarum* WCFS1 are distinctly influenced depending on the *S. aureus* strain present in the co-culture, reflecting the specific interactions and physiological adjustments in response to each strain.

#### 3.3.2. Functional Analysis of Differentially Expressed Proteins from *L. plantarum* WCFS1 During Co-Culture with *S. aureus* FRI-1169 and *S. aureus* MN8

To further investigate the impact of co-culture on *L. plantarum* WCFS1, we analyzed the functional roles of differentially expressed proteins in both the spent medium and cytosol (Supplementary Table S3). These proteins offer insight into *L. plantarum*’s physiological state and its potential influence on *S. aureus*. In the spent medium from the co-culture with *S. aureus* FRI-1169, proteins such as ArgR (involved in nitrogen regulation), CelC (carbohydrate metabolism), and OppC (peptide transport) were identified, suggesting that *L. plantarum* releases proteins associated with nutrient acquisition. Proteins like HslO (a heat shock protein) and SrtA (involved in cell wall synthesis) hint at interactions affecting protein stability and cell wall integrity in *S. aureus* FRI-1169. In contrast, during co-culture with *S. aureus* MN8, proteins like VanR (related to cell wall stability) and MvaA (involved in isoprenoid biosynthesis) were differentially expressed, indicating a potential impact on *S. aureus* MN8′s membrane integrity. Additionally, proteins like ClpC (protein quality control) and FolP (folate biosynthesis) suggest effects on stress responses and metabolic functions, while the upregulation of GalE (carbohydrate metabolism) points to shifts in nutrient utilization. In the cytosol, *L. plantarum* WCFS1 exhibited the expression of proteins like CopR (involved in copper resistance) and CydA (linked to respiratory processes) during co-culture with *S. aureus* FRI-1169, suggesting a focus on oxidative stress management and metabolic balance. During co-culture with *S. aureus* MN8, proteins such as ArgH (involved in arginine biosynthesis) and MsrA (responsible for repairing oxidative damage) were differentially expressed, indicating adaptive responses related to amino acid metabolism and oxidative stress repair. These findings underscore the distinct metabolic and stress responses by *L. plantarum* to the two different strains of *S. aureus*, highlighting its ability to modulate its physiological processes to adapt to varying co-culture conditions.

## 4. Conclusions

This study provides the first comprehensive proteomic analysis of interactions between *Lactobacillus plantarum* WCFS1 and *Staphylococcus aureus* strains FRI-1169 and MN8, indicating a directional and strain-specific antagonism. *L. plantarum* exhibited a stronger impact on *S. aureus*, with more differentially expressed and upregulated proteins, while *S. aureus* strains responded with fewer DEPs and more downregulated proteins. The results demonstrate that *L. plantarum* influences key metabolic, regulatory, and stress response pathways in *S. aureus*, highlighting its potential as a non-antibiotic biocontrol agent. Furthermore, strain-specific differences in *S. aureus* responses suggest complex bacterial dynamics during co-culture. These findings underscore the effectiveness of proteomic analysis in informing new hypotheses and finding ways to manage *S. aureus* infections without the use of antibiotics or actually enhance antibiotic efficacy.

## Data Availability

The original contributions presented in the study are included in the article/Supplementary Material, further inquiries can be directed to the corresponding author.

## Supplementary Materials

The following supporting information can be downloaded at: https://www.mdpi.com/article/10.3390/microorganisms12122432/s1. Supplemental Table S1. Lists of proteins related to antibiotic resistance, heavy metal resistance, and virulence factors identified in the spent medium (Sheets 1 to 3) and cytosol (Sheets 4 to 6) of *L. plantarum* WCFS1 and *S. aureus* FRI-1169 (Sheets 7 to 9 and 10 to 12, respectively) during co-culture with each other. Supplemental Table S2. Lists of proteins related to antibiotic resistance, heavy metal resistance, and virulence factors identified in the spent medium (Sheets 1 to 3) and cytosol (Sheets 4 to 6) of *L. plantarum* WCFS1 and *S. aureus* MN8 (Sheets 7 to 9 and 10 to 12, respectively) during co-culture with each other. Supplemental Table S3. A list of proteins of *L. plantarum* WCFS1 during co-culture. In the spent medium, a total of 41 proteins (Sheet 1) and 67 proteins (Sheet 2) were differentially expressed in co-culture with *S. aureus* FRI-1169 and MN8, respectively, with 314 proteins expressed in common (Sheet 3). In the cytosol, 173 proteins (Sheet 4) and 34 proteins (Sheet 5) were differentially expressed in co-culture with *S. aureus* FRI-1169 and MN8, respectively, with 1155 proteins expressed in common (Sheet 6).
